# Supplementary material for: Dynamic transcriptomic profiles of zebrafish gills in response to zinc supplementation
Source: BMC Genomics. 2010 Oct 11;11:553. doi: 10.1186/1471-2164-11-553 (PMC3091702; doi:10.1186/1471-2164-11-553)
Supplement: Additional file 2 — Interactive Direct Interaction Network representing the molecular interactions between zinc, copper, iron, calcium and proteins encoded by transcripts changed by zinc supplementation. Mini web-site containing index.html and hyperlinked pages in subdirectory describing a Direct Interaction Network automatically generated based on curated interactions contained within the proprietary PathwayArchitect database. Ovals represent proteins and the circles symbolize metal ions. Objects are coloured by their abundance in zebrafish at the time-point they were significantly different from the control is a scale from -4 fold (dark green) to +4 fold (dark red). Where significant differences were found at more than one time-point, the colour overlay shows expression at the first instance. Dark blue squares denote 'binding', and light blue squares 'expression'; green squares stand for 'regulation', green diamonds for 'metabolism', and green circles for 'promoter binding'. Arrow heads indicate directionality of the interaction where annotated. All nodes and edges can be further interrogated by selecting the relative area of the image. [file 1471-2164-11-553-S2.zip › PathwayArchitect Zn xs DIN/98165.html]

# PROTEIN: OTX2

|  |  |
| --- | --- |
| Name | OTX2 |
| Type | PROTEIN |
| Description | orthodenticle homolog 2 (Drosophila) |
| Note | This gene encodes a member of the bicoid sub-family of homeodomain-containing transcription factors. The encoded protein acts as a transcription factor and may play a role in brain and sensory organ development. A similar protein in mice is required for proper forebrain development. Two transcript variants encoding distinct isoforms have been identified for this gene. Other alternative splice variants may exist, but their full length sequences have not been determined. |
| Alias | Homeobox protein OTX2 |
|  | OTX2 |
|  | Otx2 |
|  | E130306E05Rik |
|  | Otx-2 |
|  | orthodenticle 2 |
|  | orthodenticle homolog 2 |
|  | homeobox protein OTX2 |
|  | MGC45000 |


---

|  |  |
| --- | --- |
| GO Component | nucleus |


---

|  |  |
| --- | --- |
| GO ID | GO:0009952 |
|  | GO:0003677 |
|  | GO:0008589 |
|  | GO:0030901 |
|  | GO:0006355 |
|  | GO:0042706 |
|  | GO:0030528 |
|  | GO:0006357 |
|  | GO:0005515 |
|  | GO:0003700 |
|  | GO:0045165 |
|  | GO:0005634 |
|  | GO:0030182 |
|  | GO:0009953 |
|  | GO:0030154 |
|  | GO:0007417 |
|  | GO:0008190 |
|  | GO:0009887 |
|  | GO:0030900 |
|  | GO:0006350 |
|  | GO:0001708 |
|  | GO:0007275 |
|  | GO:0045449 |
|  | GO:0040036 |


---

|  |  |
| --- | --- |
| MIM | MIM:600037 |


---

|  |  |
| --- | --- |
| Connectivity | 76 |


---

|  |  |
| --- | --- |
| Entrez ID | 18424 |
|  | 5015 |


---

|  |  |
| --- | --- |
| Agilent ID | A\_23\_P48663 |
|  | A\_14\_P136915 |
|  | A\_52\_P49080 |
|  | A\_14\_P105885 |
|  | A\_51\_P250191 |
|  | A\_14\_P201904 |


---

|  |  |
| --- | --- |
| Cellular Localization | Nucleus |
|  | Organelle |
|  | Cell |


---

|  |  |
| --- | --- |
| Pathway | Zn xs inventory |
|  | Zn xs DIN |


---

|  |  |
| --- | --- |
| GO Process | cell differentiation |
|  | cell fate specification |
|  | regulation of smoothened signaling pathway |
|  | regulation of transcription |
|  | neuron differentiation |
|  | forebrain development |
|  | eye photoreceptor cell fate commitment |
|  | organ morphogenesis |
|  | cell fate commitment |
|  | anterior/posterior pattern formation |
|  | dorsal/ventral pattern formation |
|  | regulation of transcription, DNA-dependent |
|  | regulation of transcription from RNA polymerase II promoter |
|  | transcription |
|  | central nervous system development |
|  | regulation of fibroblast growth factor receptor signaling pathway |
|  | development |
|  | midbrain development |


---

|  |  |
| --- | --- |
| UniGene | Hs.288655 |
|  | Mm.134516 |


---

|  |  |
| --- | --- |
| Affymetrix Probeset ID | 1425926\_a\_at |
|  | 231731\_at |
|  | 242128\_at |
|  | 53326\_at |
|  | Hs.288655.0.S1\_3p\_at |
|  | Hs.30837.0.A1\_3p\_at |
|  | RC\_H08155\_at |
|  | TC17915\_at |
|  | TC17915\_g\_at |


---

|  |  |
| --- | --- |
| GO Function | eukaryotic initiation factor 4E binding |
|  | protein binding |
|  | transcription factor activity |
|  | transcription regulator activity |
|  | DNA binding |


---

|  |  |
| --- | --- |
| Nucleotide | BC017609 |
|  | NM\_172337 |
|  | AK081535 |
|  | U96488 |
|  | BC027104 |
|  | AK087527 |
|  | AF093138 |
|  | X68884 |
|  | BE781530 |
|  | NM\_021728 |
|  | AF298117 |
|  | BC032579 |
|  | AK053741 |
|  | NM\_144841 |
|  | BC029667 |
|  | AB037505 |


---

|  |  |
| --- | --- |
| Protein | BAC39913 |
|  | CAA48755 |
|  | AAH32579 |
|  | AAH29667 |
|  | P80206 |
|  | NP\_758840 |
|  | BAC38249 |
|  | AAG16243 |
|  | NP\_068374 |
|  | P32243 |
|  | BAA90425 |
|  | AAB57690 |
|  | AAH17609 |
|  | BAC35500 |
|  | AAH27104 |
|  | AAD31385 |
|  | NP\_659090 |


---

|  |  |
| --- | --- |
| Organism | Mammal |


---

|  |  |
| --- | --- |
| Location | chromosome 14, 14q21-q22 (Homo sapiens) |
|  | 14 19.0 cM (Mus musculus) |
|  | chromosome 14, 14 19.0 cM, 14 C1 (Mus musculus) |


---

|  |  |
| --- | --- |
